# Recurrent episodes associated with childbearing: a matrix of associations

**DOI:** 10.1007/s00737-016-0681-x

**Published:** 2016-10-17

**Authors:** Ian Brockington

**Affiliations:** Lower Brockington Farm, University of Birmingham, Bredenbury, Bromyard, Herefordshire HR7 4TE UK

**Keywords:** Prepartum onset, Early postpartum onset, Late postpartum onsets, Post-abortion onset

## Abstract

A study of several hundred recurrent puerperal psychoses shows that about half of those with known onset recur in the same phase of reproduction, and half have onsets in different phases. Onsets in the same phase are especially a feature of prepartum psychosis and are the strongest indication of a specific trigger operating during pregnancy. Onsets in different phases provide a *prima facie* case for links between ‘puerperal psychosis’ and other reproductive onsets. They suggest that the ‘picture puzzle’ is not just about early onset puerperal psychosis, but a group of related reproductive triggers.

## Introduction

It has been known since the sixteenth century that puerperal psychoses can recur. The literature contains 450 examples of mothers with multiple episodes.

The knowledge that a substantial proportion of mothers will suffer a recurrence in the next and subsequent pregnancy has been used as a strategy to study the pathogenesis of psychosis and explore causal hypotheses (Wieck et al. 1991; Meakin et al. 1995). But there is much more that can be learned from recurrent episodes.

The published cases fall into three groups—those with no information on onset, those with onset in the same time frame, and those with onsets at different phases of the reproductive process:

- When there is no information on onset, multiple episodes merely support the general tendency to recur, which is the best-founded and most widely known fact about puerperal psychosis
- When episodes begin in the same time frame, it supports the existence of a specific causal factor, acting at that stage of the reproductive process; this becomes more convincing if the mothers are followed long term, and their episode rate is known, and low enough to exclude sporadic attacks
- When mothers have multiple episodes with onsets during different phases, this is evidence of the interdependence of triggers

This paper will explore the data on recurrent cases published in the literature.

## Method

### Omissions

From 450 recurrent cases, the following were omitted.

- Those in which all episodes were of unknown onset
- Those with only one episode of known onset
- Those in which the other episodes were organic, post-operative or non-psychotic

This left 265 for analysis, of which 133 had episodes limited to one time frame and 132 had episodes starting in different time frames. These were used to construct a ‘matrix of associations’.

### The matrix of associations

Five time frames are used:

- Onset within 3 months of an abortion (miscarriage, termination, extra-uterine pregnancy or hydatidiform mole)
- Onset during the 9 months of pregnancy
- Early postpartum onset (before the 21st day after the birth)
- 4-13-week onset (the last 10 weeks of the first trimester)
- Late postpartum onset (the remaining 9 months of the first postpartum year).

Weaning onset was not included because of the small number of cases.

Cases were assigned to boxes when a mother had one or more episodes in both time frames. Thus, a mother with two early and three 4-13-week onsets scored just one in the box for associated early and 4-13-week onsets, but a mother with onsets 3 weeks, 3 and 4 months after the birth counted one in each of three boxes—early & 4-13-week onsets, early & >3-month onsets and 4-13 week & >3-month onsets.

The last column shows the number of published cases with onsets in that time frame.
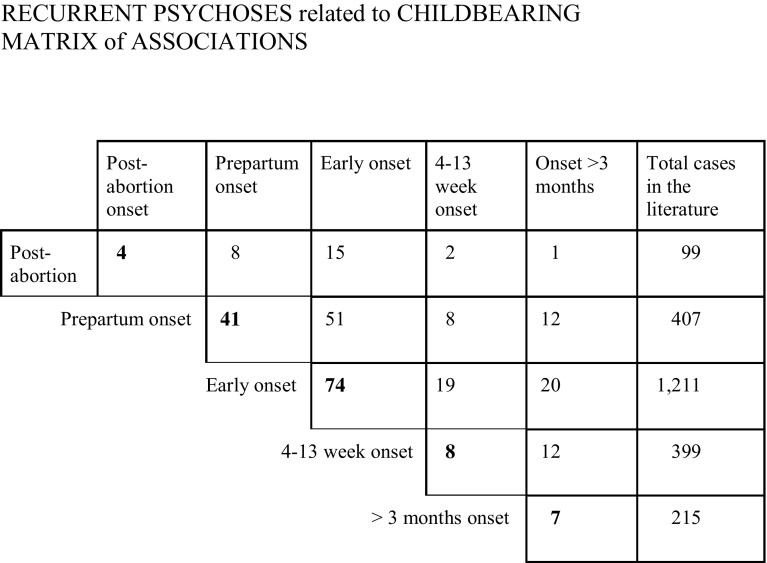


### Recurrences all in one time frame

#### Prepartum onset

If the numbers are related to the totals in the last column, prepartum onsets have the highest proportion (10 %). This substantiates the surprisingly large number of serial prepartum cases reported in the literature. Five authors made general statements reporting four (Jolly 1911), five (Esquirol 1838; Clouston 1896), seven (Knauer 1897; Combs 1956), nine (Friedreich 1835) or twelve (Menzies 1893) prepartum episodes, without giving details. Another 30 had details on timing: 14 had all onsets in one trimester (six in the 1st trimester, four in the 2nd trimester and four in the 3rd trimester). To these can be added six cases with at least two prepartum episodes, as well as postpartum episodes (Marcé 1858; Boudrie 1878; Grosse 1968; Durand et al. 1978; Luauté et al. 1991; Mendhekar et al. 2002; Kleinsman and Knoppert-van de Klein 2006).

The tendency for prepartum psychosis to recur is the best evidence for the existence of a specific prepartum trigger for non-psychotic episodes. Stronger evidence requires patients followed long term, so that the frequency of psychotic episodes can be estimated. There are five in the literature. The probability that the prepartum episodes were not sporadic was calculated from the length of study, number of pregnancies and abortions and number of episodes unrelated to childbearing, using Fisher’s exact test. In three, it was in the range 0.06–0.09 (Cortyl 1877; Grosse 1968; Mendhekar et al. 2002). These are the other two (Elfes 1912; Durand et al. 1978):

A 25-year old single woman gave birth to two children, both of whom died within 10 weeks. Pregnant for the 3rd time, she became depressed and wanted to drown herself. Admitted to hospital, she was restless, talked day and night and thought that knocking on the window meant that her two children were still alive; later she developed catalepsy and heard voices saying she would be killed (1st prepartum episode). After the birth she recovered. The following year she became anxious about another pregnancy, and started running about aimlessly, ringing her hands. Admitted to hospital, she was making stereotypic movements, hopping like a frog, weeping and singing, and smearing faeces; she heard voices and was intermittently energetic and over-active. She recovered and had a miscarriage (2nd prepartum episode). After some months she became pregnant again. She had two seizures and was unconscious for a time. Admitted to hospital she laughed, sang and wept, talked monotonously with flight of ideas, saw ghosts, tore her clothes, and awaited investigation by the police. She expected to be married, but, after a letter to the boyfriend was returned undeliverable, became disturbed and aggressive, wanted to strangle herself and ran about aimlessly. At 4 months gestation, the pregnancy miscarried, but the illness continued, with restlessness, shouting and cursing, stereotypies, obscenity and aggression to the nurses (3rd prepartum episode). In the next 4 years she had two similar episodes unrelated to pregnancy.

In 7 years she had one full pregnancy, two miscarriages and two unrelated episodes; all three pregnancies were associated with psychotic episodes requiring hospital admission (*p* = 0.03). All episodes had onset in the 1st trimester, which is more improbable (*p* = 0.004). But the social circumstances were so adverse that an alternative diagnosis of pseudo-psychosis—a series of emotional crises—is just possible:

A mother of two became pregnant for the 3rd time. In the 1st trimester, she developed an acute psychosis with manic features. During the next 6 years she had two similar acute episodes. She became ill again when 2 months pregnant (no details), and again in the 9th month; this continued and a few days after the birth she presented with insomnia, labile mood, hyperactivity, a confusional-delirious picture and phases of mutism. She had audio-visual and caenesthetic hallucinations and memory difficulties: she mistook the sex of the baby; she felt the baby inside her, and the pains of labour. Some days later her menses reappeared and she immediately recovered with complete amnesia for the episode.

This mother had two full pregnancies and five episodes in 8 years—three prepartum (one of which continued after the birth) and two unrelated; for prepartum episodes, *p* = 0.04, only just significant statistically.

The calculations are only approximate and there are difficulties in defining episodes; these two cases support the presence of a specific prepartum factor, but there is a need for more cases, with a longer period of observation.

#### Early postpartum onset

There are many examples of serial onsets within the first 3 weeks postpartum; eight mothers had three onsets in this time frame, and two had brief details of four episodes (Bell 1833; Hadley 1941). There are impressive examples of mothers who suffered only from early postpartum onsets, with long periods of normal mental health. For example, this mother (Bonse 1989) had three early psychoses after four births and five unrelated episodes in 23 years (*p* < 0.0001).

A woman, whose brother suffered from manic episodes, became depressed at the age of 15, coincident with ‘diminished menses’. At the next menses she was worse, and believed the police were after her. Admitted to hospital, she was perplexed, and complained that everything seemed strange and threatening. At 20 she gave birth to her 1st child. On day 3 she became febrile, restless and irritable. She seemed depressed and said she felt anxious ‘as in war’. She noticed strange smells and expressed ideas of persecution and poisoning. Her speech was incoherent with flight of ideas, and she may have had auditory hallucinations. She recovered after 4 weeks in hospital. Two years later she gave birth to her 2nd child. Within 2–3 days she became overactive with elevated mood and incoherent speech and behaviour; she was running about at night, and making senseless purchases. After 2 months she switched to depression and thought her husband should shoot her. Four months after the birth she recovered. Eighteen months later she gave birth to her 3rd child, who died some hours later; there was no recurrence, but in the next year she suffered an episode unrelated to childbearing, with depression, mutism, a suicide attempt and hypomania. At 26 she gave birth to her 4th child. On day 18 another episode began with depression swinging to hypomania, for which she was hospitalized for 6 months. In the next 12 years she had three more unrelated bipolar episodes.

There are several similar cases:

- A mother (Gödtel 1979) had two episodes after three births and no unrelated episodes in 17 years (*p* = 0.007)
- A mother (Boutet 1913) had two 8-day onsets, three unaffected births and only one further episode 39 years after the first (*p* = 0.003)
- A mother (Fallgatter et al. 2002) had three pregnancies in 15 years, with four episodes (days 11 and 7 postpartum, at 24 weeks gestation and day 9 after completion of the same pregnancy); she remained well for 15 years (*p* < 0.0001).

A series of women with post-menopausal episodes (Robertson Blackmore et al. 2008) included two who suffered early postpartum episodes after both their births and no other episodes for 20 years. All this is strong evidence for an early postpartum trigger, but it is unnecessary because this has already been established by epidemiological surveys.

### Late postpartum onsets

There are eight cases in the literature with two 4-13-week onsets and no other reproductive episodes. One mother had three episodes, but only a vague description—“She became insane some weeks later, each time with various delusions” (Gilmore 1892). In addition to those with multiple episodes limited to this time frame, one mother had seven postpartum episodes, including three late onsets—at 4 weeks, 2 and 3 months, with the rest of unknown onset (Ideler 1851). A Danish patient (Holm 1874) had three early and three 4-13-week postpartum onsets, as well as four unrelated episodes in 104 non-reproductive trimesters (*p* = 0.0001); but the postpartum episodes started in two separate time frames.

As for episodes with onset more than 3 months after the birth, there are a surprisingly large number (seven) with two or more episodes (Hurt 1911; Masieri 1925; Mitkus 1927; Rabinowitsch 1928; Ménaché 1929; Fumarola 1935; Blinov et al. 1936). One mother (Ménaché 1929) had four episodes of acute mania with onset 2, 5, 6 and 6 months after her four births and no other episodes in 8 years (*p* = 0.03). Two with brief recurrent hallucinatory episodes (Hurt 1911) have been summarized in another paper (Brockington in press). These recurrent cases, with two to five episodes each, support the action of some unknown but specific late postpartum factor.

### Post-abortion onset

This onset group had the smallest number of cases with episodes limited to that time frame. The first hint was a telegrammatic mention in mediaeval Latin (de Berger 1745):

I have learned of a 20-year old, who suffered, like her mother, 4 months after her last menstrual period: she twice emitted a foetus and suddenly became insane, but it did not last long.

Only three other cases have been reported (Bartens 1884; De Gorsky 1888), one of them in an unpublished master thesis (Roldan 1994). But others, who had postpartum episodes as well, had more than one post-abortion episode, including two post-abortion and one postpartum episode (Capelle 1929) and two post-abortion and four postpartum episodes (Mahe et al. 1999). The most impressive is this case, published by two separate authors (Steinmann 1935; Schwingenheuer 1953):

A 25-year old, whose brother had a diagnosis of schizophrenia, had a puerperal episode @ 21, and post-abortion episodes @ 22, 23 and 24, each lasting about 3 months and marked by confusion and flight of ideas. She was admitted for another episode 5 weeks after a 4-month miscarriage: she manifested silly euphoria, unmotivated laughter, unstoppable pressure of speech, incoherence, verbigeration and hand-clapping. Schwingenheuer reported only three post-abortion episodes, but a further unrelated episode at the age of 30.

She had three or four post-abortion episodes and one postpartum episode in 9 years, during which she had only one (reported) non-reproductive episode. This would be highly significant statistically (*p* = 0.0002), but Steinmann’s description was limited to a schema, without narrative details, and Schwingenheuer’s was also brief.

### Onset in different time frames

All associations between onset groups had at least five examples, except the association between post-abortion and both groups of late postpartum onsets. There is, therefore, a *prima facie* case for a general factor underlying all these onsets. This could be a shared diathesis (such as the bipolar/cycloid diathesis) or shared element(s) in the trigger or pathogenesis. The number of cases with both early and prepartum onsets (51) is impressive—more than twice those with both early and late postpartum onsets (19, combining the two groups), although there were 614 late postpartum onsets in the literature and only 407 prepartum cases; this suggests that the early onset and prepartum triggers have more in common. The large number of mothers, who suffered recurrent reproductive episodes in different time frames, is evidence that the ‘picture puzzle’ is not limited to early onset puerperal psychosis, but to a group of related reproductive triggers.

The findings from the literature were confirmed by my own series, which had over 100 eligible mothers with recurrent episodes. All associations had at least three examples except post-abortion and late postpartum (3–12 months) onsets (Brockington 2014).

When considering the possibility that the association between all onset groups is due to a shared diathesis, not a shared trigger, the baseline frequency of episodes unrelated to reproduction, determined in mothers followed long term, is germane. To address this question, one needs cases with diverse reproductive onsets and no unrelated episodes, studied for many years. There are only a few relevant cases in the literature:

A mother (Kogerer and Pawlicki 1934), followed for over 10 years, had one post-abortion, one prepartum and no unrelated episodes:

A 26-year old was first admitted in the 8th month of her 1st pregnancy, with 8 days history of anxious confusion; she had made three suicide attempts. Her 2nd admission was 7 years later, 8 days after curettage for an incomplete abortion. She was confused with catatonic features.

Another mother (Bonse 1989), followed for 20 years, suffered from episodes related to weaning, menstruation, childbirth, short gestation and pregnancy, as well as unrelated episodes:

A 28-year old, with a strong family history of mental illness, gave birth to her 1st child, and breast-fed for 4 months. After weaning she developed insomnia and restlessness. Her ideas were lively and she acted on a fear that the house was burning down; she also thought she was under surveillance because of her poor child-care. Admitted to hospital, she was perplexed and confused and had difficulty in distinguishing dreams from reality; she misidentified people and had a persistent idea that she was pregnant. She improved, relapsed and then recovered, but had several premenstrual deteriorations. Three years later she gave birth to her 2nd child. On day 3 she became sleepless and anorexic with pathological ideas of guilt. Admitted to hospital she was perplexed, agitated and depressed with ‘paranoid-hallucinatory elements’. Discharged after 2 months, she became hypomanic, spent a lot of money and gave presents to everybody. A year later she became pregnant for the 3rd time. In the 2nd trimester she became depressed, then manic. She heard voices - the neighbours were talking about her, and God and his angels were protecting her. Admitted to hospital, she was anxious and retarded and had difficulty with her memory. She was instructed by good and bad voices. She gave birth to a 5-month macerated foetus. Two weeks after improvement she relapsed with hypomania. She had three unrelated episodes.

Finally, there is this mother, one of those comprehensively described in a superb Dutch thesis (Van Steenbergen-van der Noordaa 1941); she was followed for 39 years, during which she suffered an untimed postpartum episode, an eclamptic episode, one with onset 4–13 weeks after childbirth, one shortly before parturition and only two unrelated episodes:

A 27-year old was admitted to an asylum for 9 months following the birth of her 2nd child. Two years later she gave birth to her 4th child, after which she suffered a seizure, associated with albuminuria and oedema of the feet. The child soon died. She became disturbed - laughing, singing, restless, disorientated, with visual and auditory hallucinations, stereotypies and catalepsy. She recovered 18 months later. Nine weeks after her 5th delivery she started to sing and pray. Her speech was disturbed with flight of ideas and neologisms. She was restless and excited. She wanted to visit her mother, dead these 2 years. She seemed to be in a dream-like state. She recovered in 4 months. At the age of 36 she became ‘confused’ at the end of her 7th pregnancy – she was praying, had dead people in her mind, and 2 days before admission ran away from home and took a carriage to a celebration; she was somewhat elevated in mood, cheered the doctor, and had fits of laughing. Five years later she again developed hyperactivity with pressure of speech, singing and dancing, preoccupied with religion, visions of the Virgin Mary and auditory hallucinations. On admission she was cataleptic. She recovered in 2 months, and remained well for 11 years, when she became overactive and excited, spoke nonsense and tried to jump from a 2nd floor balcony. From that time she remained in hospital, with bizarre ideas, laughing attacks and grimacing until her death from cancer at the age of 66.

## Discussion

The study of recurrent childbearing psychoses reveals large numbers of mothers with a bewildering array of complex associations. This paper focuses on reproductive onsets (prepartum, early and late postpartum, weaning and post-abortion), but in addition there is much evidence that all these onsets are also associated with menstrual psychosis (Brockington 2014, 2016).

A limitation is that the evidence comes not from epidemiological surveys, which can only detect the most potent trigger (early postpartum), but from a study of published cases, followed long enough to give information on the natural history of the disease. Furthermore, the findings are based on a literature review by a single observer. They need confirmation by other clinicians, following a large series of mothers long term, with full verbatim descriptions of episodes, and using 2-rater consensus diagnoses. There are many consultants in various European countries with extensive clinical experience, who will be in a position to confirm or refute these findings. A replication, using a rigorous methodology, would generate data worthy of statistical examination and allow a more detailed exploration of these associations. Such a study would be worthwhile, because the ‘matrix of associations’ raises fundamental questions about the nature of the ‘picture puzzle’ (Paffenbarger 1961), and shared elements in the triggering of reproductive episodes.
